# Novel *LIPC*-related recessive form of postural proprioceptive deficits in Brown Swiss cattle

**DOI:** 10.1016/j.vas.2026.100645

**Published:** 2026-04-02

**Authors:** Bettina A. Weber, Aline Zimmermann, Irene M. Häfliger, Franz R. Seefried, Mireille Meylan, Cord Drögemüller, Joana Jacinto

**Affiliations:** a Institute of Genetics, Vetsuisse Faculty, University of Bern, 3012 Bern, Switzerland; b Qualitas AG, 6300 Zug, Switzerland; c Clinic for Ruminants, Vetsuisse Faculty, University of Bern, 3012 Bern, Switzerland

**Keywords:** Bovine, Genetic disorder, Selection, Rare disease, Mendelian, Lipid metabolism

## Abstract

Genetic neuromuscular disorders (NMDs) are characterized by progressive skeletal muscle degeneration and weakness. In Brown Swiss (BS) cattle, three recessive variants have been associated with NMDs. This study aimed to (1) describe the phenotype of BS cattle affected by a novel form of postural proprioceptive deficits, (2) identify a candidate genetic variant using whole-genome sequencing (WGS), and (3) estimate its prevalence in the Swiss BS population. A first BS heifer (case 1) showing ataxia underwent clinical and hematological examination, followed by WGS of the heifer and its sire. Variants were filtered and compared against 5577 controls. Candidate variants were evaluated *in silico* for predicted pathogenicity, genotyped in national breeding cohorts, and clinically assessed in additional homozygous animals. A rare homozygous missense variant in *LIPC* (chr10:51715800G>C; NM_001035410.1:c.924C>G; p.Phe308Leu; omia.variant:1842) was identified and *in-silico* predictions classified this variant as deleterious. Population-level genotyping of over 20.000 BS cattle revealed a variant allele frequency of 17% with significant deviation from the Hardy–Weinberg equilibrium (p-value=1.14 × 10⁻^20^), suggesting possible lethality in homozygotes. Case 1, as well as 11 additional *LIPC*-homozygous BS females (mean age 2.4 ± 0.2 years), exhibited consistent neurological signs, including postural deficits and subconscious proprioceptive ataxia. Biochemical profiles revealed hypercholesterolemia, hypertriglyceridemia, and decreased HDL cholesterol, suggesting dyslipidemia. Pedigree analysis identified a shared ancestor and inbreeding loops, supporting recessive inheritance. This study describes a novel autosomal recessive *LIPC*-related juvenile-onset NMD in BS cattle characterized by postural proprioceptive deficits with a tendency towards dyslipidemia. More research is needed to better understand the effects of the identified variant and prove that it is the cause.

## Highlights

• A recessive *LIPC* variant was identified in Brown Swiss cattle with neurological signs.
• Affected cattle show postural proprioceptive deficits and a tendency toward dyslipidemia.
• Pedigree information of affected cattle suggests recessive inheritance.
• *LIPC* variant found in Brown Swiss cattle with an allele frequency of 17.%.
• Including the *LIPC* variant in selection is key to reducing disease incidence.

## 1 Introduction

Genetic neuromuscular disorders (NMDs) comprise a heterogeneous group of conditions affecting several mammalian species that can present congenitally or later in life (Nicholas et al., 2025). NMDs are characterized by progressive muscle degeneration and weakness, affecting skeletal muscle function (Zatz et al., 2016). The nervous system is also susceptible to changes in metabolic homeostasis and impairments can lead to NMDs (Mayhew & Mackay, 2022).

In cattle, 22 NMDs have a known molecular cause (Nicholas et al., 2025). In particular, three recessively inherited NMDs are known to occur in Brown Swiss (BS) cattle: bovine progressive degenerative myeloencephalopathy (BPDME) *PNPLA8*-related that is also known as Weaver Syndrome (OMIA: 000827-9913)(Kunz et al., 2016), spinal muscular atrophy (SMA) *KDSR*-related (OMIA: 002390-9913)(Krebs et al., 2007), and spinal dysmyelination (SDM) *SPAST*-related (OMIA: 001247-9913)(Thomsen et al., 2010). The Original Braunvieh (OB) population is the ancestor of the world-renowned BS population and does not carry these NMD-causing alleles (Glatthard et al., 2025). We recently reported that through selective breeding, these deleterious alleles have been largely eliminated from the current Swiss BS population (Glatthard et al., 2025).

BPDME has a juvenile onset, with initial clinical signs typically appearing between 5 and 8 months of age (Kunz et al., 2016; Stuart & Leipold, 1983). The initial clinical signs include bilateral pelvic limb weakness, which progresses to ataxia, decreased proprioceptive reflexes and dysmetria of the pelvic limbs (Stuart & Leipold, 1983). Ultimately, the condition leads to euthanasia due to permanent recumbency, occurring 12 to 18 months after the onset of clinical signs (Stuart & Leipold, 1983). In histopathology, lesions are confined to the white matter and consistent with spinocerebellar degeneration, including axonal swelling and degeneration, loss of axons and myelin, and status spongiosus (El-Hamidi & Leipold, 1990; Stuart & Leipold, 1985).

SMA presents with a variable onset, ranging from the neonatal period to approximately six weeks of age (El‐Hamidi et al., 1989; Krebs et al., 2007; Stocker H et al., 1992). Affected calves initially display pelvic limb weakness, which rapidly progresses to severe appendicular muscle atrophy, reduced lower limb reflexes, and quadriparesis, ultimately leading to euthanasia due to permanent recumbency (El‐Hamidi et al., 1989; Stocker et al., 1992). The main histopathological findings consist of neurogenic muscle atrophy, chromatolysis, and degeneration of somatic motor neurons (El‐Hamidi et al., 1989).

SDM is a congenital NMD characterized by permanent lateral recumbency, accompanied by opisthotonus, stiff limb extension, skeletal muscle atrophy and tremors (Agerholm et al., 1994; Hafner et al., 1993). Affected calves typically are euthanized within the first month of life (Agerholm et al., 1994). Histopathological findings include moderate systemic skeletal muscle atrophy and severe bilateral symmetric hypomyelination with partial demyelination of cervical and thoracic spinal cord segments (Agerholm et al., 1994; Hafner et al., 1993).

In this context, the present study aimed to 1) characterize the clinical presentation of a novel form of postural proprioceptive deficits in BS cattle, 2) identify the causative genetic variant using whole-genome sequencing (WGS), and 3) determine its prevalence in the Swiss BS population to inform strategies for disease prevention at population level. We hypothesize that this novel form of postural proprioceptive deficits has a genetic molecular cause and segregates within the BS population, and that its identification and characterization will provide insights relevant to disease prevention.

## 2 Material and methods

### 2.1 Ethics statement

All animals in this study were examined with the consent of their owners and handled in accordance with the Swiss Animal Welfare Act and institutional ethical guidelines (Swiss Federal Food Safety & Veterinary Office, 2024). The SNP array genotypes were generated during population-wide genotyping of Swiss dairy populations for the purpose of genomic selection. Collection of blood samples was approved by the Cantonal Committee for Animal Experiments (Canton of Bern; permit BE94/2022).

### 2.2 Index case clinical investigation

The index case, a 2.5 -year-old BS heifer (case 1), was referred to the Clinic for Ruminants of the University of Bern for gait abnormalities consistent with ataxia and resembling BPDME. Both the sire and dam were reported to not show any clinical signs. A complete clinical examination of case 1 was performed including a general clinical examination and a neurological clinical assessment. Additionally, complete blood count (CBC), plasma biochemistry and cerebrospinal fluid analysis were performed.

### 2.3 DNA extraction

The genomic desoxyribonucleic acid (DNA) was isolated from EDTA-blood of case 1 and from semen of the sire. The DNA was extracted using the Promega Maxwell RSC DNA System (Promega, Dübendorf, Switzerland).

### 2.4 PCR and Sanger sequencing of the PNPLA8 variant

To evaluate the presence of the *PNPLA8* (XP_005205501.2:p.Ser568Asn) variant that was previously reported in BPDME-affected BS cattle, genotyping by PCR and Sanger sequencing was performed in case 1 as previously reported (Kunz et al., 2016).

### 2.5 Whole-genome sequencing and variant calling

WGS data was generated using the Illumina NovaSeq6000 (Illumina Inc., San Diego, CA, USA) on the genomic DNA extracted from samples of case 1 and its sire. The sequenced reads were mapped to the ARS‐UCD1.2 reference genome (Rosen et al., 2020), resulting in an average read depth of 21.2 ×, and single-nucleotide variants (SNVs) and small indel variants were called. The applied software and steps used to process FASTQfiles into Binary Alignment Map (BAM) and Genomic Variant Call Format (GVCF) files were in accordance with the processing guidelines of run 7 of the 1000 Bull Genomes Project (Hayes & Daetwyler, 2019), except for the trimming, which was performed using fastp (Chen et al., 2018). Downstream processing of the genomic data was performed as reported previously (Häfliger et al., 2020). The effects of all called variants were functionally evaluated with snpEff v5.0c (Cingolani et al., 2012), using the NCBI Annotation Release 106 (National Library of Medicine, 2018). This resulted in the final GVCF file, comprising jointly genotyped individual variants and their functional annotations.

To identify variants associated with the described phenotype, the genotype of case 1 was compared to 1037 cattle genomes of different breeds including 167 BS cattle sequenced as part of the ongoing Swiss Comparative Bovine Resequencing project. The variant filtering was performed under the following hypotheses: 1) a recessive inheritance, where case 1 was homozygous, the sire an obligate heterozygote, and the controls were either heterozygous or homozygous wildtype, and 2) a spontaneous dominant *de novo* mutation, where case 1 was heterozygous and the sperm DNA of the sire was either homozygous wildtype or mosaic heterozygous and the controls were homozygous wildtype.

### 2.6 Occurrence of variants in a global control cohort

The comprehensive variant catalogue from run 9 of the 1000 Bull Genomes Project was available to investigate the allelic distribution of the found variants within a global control cohort (Hayes & Daetwyler, 2019). That dataset consisted of 5116 bovine genomes from a wide variety of >130 breeds including 293 BS cattle. This dataset included 576 cattle from the Swiss Comparative Bovine Resequencing project.

### 2.7 In silico assessment of the molecular consequences

PredictSNP1 (Bendl et al., 2014), PolyPhen-1, Polyphen-2 (Adzhubei et al., 2013), SIFT (Ng & Henikoff, 2003) and PhD-SNPg (Capriotti & Fariselli, 2023) were used to predict the biological consequences of the identified SNVs.

### 2.8 Candidate gene and candidate variant classification

The term “candidate gene” was used to describe genes based on function and/or association with NMD phenotypes in mammalian species. The term "candidate variant" was used to describe a variant deemed plausible considering the affected gene function and/or associated phenotype in mammalian species, rarity, and the predicted effect of the variant on the encoded protein, with at least two *in silico* tools predicting it to be deleterious. The variants were further classified according to the human and veterinary standards and guidelines for the interpretation of sequence variants (Boeykens et al., 2024; Richards et al., 2015). All candidate variants were visually inspected using the Integrative Genomics Viewer (IGV) (Robinson et al., 2017).

### 2.9 Target genotyping of the LIPC variant and runs of homozygosity

The identified missense variant in *LIPC* was added to the subsequently updated version of the Swiss Axiom custom genotyping arrays (Thermo Fisher Scientific, Waltham, MA, USA), designated as SWISScow (96-array layout with 314,744 markers), SWISSLD1 (384-array layout with 64,212 markers) and SWILLD1 (96-array layout with 82,277 markers), designed for genomic selection purpose in Switzerland. Thus, after four years of population-wide genotyping in Swiss dairy cattle, several hundreds to thousands of genotypes for the *LIPC* variant were available for different breeds. Subsequently, the occurrence of the three possible genotypes (homozygous, heterozygous, homozygous wildtype) was evaluated.

Genomic inbreeding coefficients were calculated from SNP genotype data following van Raden (2008) and subsequently rescaled to match the distribution of pedigree-based inbreeding coefficients; rescaled values were compared among the three possible *LIPC* genotype classes.

### 2.10 Phenotypical investigation of LIPC-homozygous Brown Swiss cattle

Based on the genotype, additional 11 *LIPC*-homozygous BS females (cases 2–12) were selected for an on farm complete clinical examination including a general clinical examination and a neurological clinical assessment. The animals examined were selected based on being alive and their owners’ consent to the inclusion in the study and clinical examination. Additionally, plasma biochemistry was performed for 5 *LIPC*-homozygous bovines. Three years after the initial investigation, follow-up interviews were conducted with the owners of five affected animals (cases 1, 4, 5, 7, and 11). The remaining owners could not be reached for a follow-up.

### 2.11 Pedigree analysis

A pedigree analysis was performed including all 12 cases that underwent a clinical examination. The pedigree graphic was obtained using the Pedixplorer (Le Nézet et al., 2025).

## 3 Results

### 3.1 Exclusion of BPDME

The PCR test determined case 1 as homozygous wildtype for the*PNLPA8* variant, therefore BPDME was excluded as a cause of ataxia.

### 3.2 Exclusion of a spontaneous dominant de novo mutation

The WGS dataset was filtered for rare heterozygous protein-changing variants in case 1. The term rare was used to describe heterozygous variants that occurred exclusively in case 1 and were absent in the control cohort excluding the sire under the assumption that a spontaneous dominant *de novo* mutation was causing the observed phenotype. The filtering revealed two rare heterozygous SNVs. However, the two variants were of uncertain significance, and neither affected a potential candidate gene (Supplementary Table S1). IGV inspection revealed that both variants were present in case 1 and were absent in the sire’s semen DNA (homozygous wildtype). These results suggest that a spontaneous dominant *de novo* mutation is unlikely to explain the observed phenotype.

### 3.3 Discovery of a recessively inherited variant in the bovine LIPC gene

**Fig. 1:**
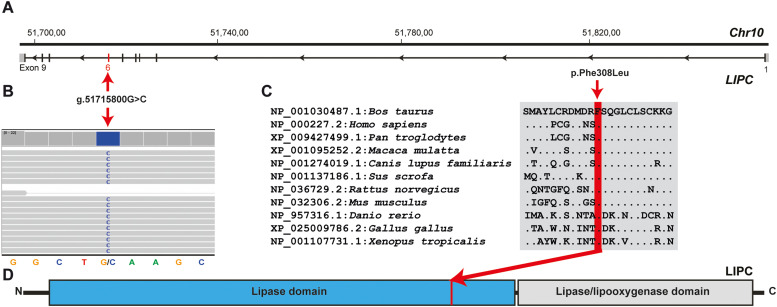
*LIPC* missense variant in a Brown Swiss heifer with postural proprioceptive deficits.

The WGS dataset was also filtered for rare homozygous protein-changing variants. This was performed under the assumption that a recessively inherited variant caused the observed phenotype. The sire of case 1 was therefore assumed to be an obligatory heterozygous carrier. This approach identified a single rare homozygous SNV with a predicted moderate impact. This was a homozygous missense variant in *LIPC* exon 6 (chr10:51715800G>C; NM_001035410.1:c.924C>G; Supplementary Table S1) and was confirmed by visual inspection on IGV (Fig. 1. A and B). The *LIPC* missense variant was predicted to change the encoded amino acid of *LIPC* residue 308 (NP_001030487.1: p.Phe308Leu), which is located in the lipase domain (Fig. 1. C and D). The *LIPC* variant was classified as pathogenic (PS1, PS4, PP1, PP3; Supplementary Table S1).

A) Structure of *LIPC* gene showing the exon 6 variant located on chromosome 10. B) Integrative Genomics Viewer screenshot presenting the Chr10:51715800G>C variant in the affected heifer. C) Cross-species sequence comparison of part of the lipase domain of the LIPC protein with the region around the p.Phe308Leu variant showing complete evolutionary conservation. D) Schematic representation of LIPC protein and its functional domains with the position of the identified pathogenic variant (red line).

### 3.4 LIPC variant occurrence in global and Swiss dairy cattle populations

Screening of the 1000 Bull Genomes Project cohort for the identified *LIPC* variant identified 80 heterozygous (ref/var) carriers, the majority of which were BS (Table 1).

**Table 1:** Occurrence of the *LIPC* missense variant.

Cohort	ref/ref	ref/var	var/var	Allele frequency
1000 Bull Genomes	5279	80*	1⁎⁎	0.008
Swiss WGS	1005	32⁎⁎⁎	1⁎⁎	0.016
SWISSCow⁎⁎⁎⁎	4442	2322	16	0.17
SWISSLD1⁎⁎⁎⁎	8951	3626	373	0.17
SWILLD1⁎⁎⁎⁎	381	149	10	0.16

⁎ Brown Swiss = 65; Unknown = 4; Modern Danish Red = 4; Finnish Ayrshire = 2; Holstein = 2; Vosgienne = 1; Latvian Brown = 1; Modern Angler = 1.

⁎⁎ Case 1.

⁎⁎⁎ All Brown Swiss.

⁎⁎⁎⁎ SNP array genotyping, all Brown Swiss Swiss WGS = Swiss Comparative Bovine Resequencing project ref = reference allele var = variant allele.

Additionally, the extensive dataset from the national genomic selection program enabled a comprehensive evaluation of the *LIPC* allele occurrence in Swiss dairy cattle across breeds. The *LIPC* variant allele segregated only in the Swiss BS population.

In the BS cohort (N = 20′270), the genotype distribution for the *LIPC* variant showed 13′774 homozygous wildtype (ref/ref), 6097 heterozygous (ref/var) and 399 *LIPC*-homozygous (var/var) animals (Table 1). The calculated allele frequency of the *LIPC* variant was 17%, and a significant deviation from the Hardy-Weinberg equilibrium was observed (p-value = 1.14 × 10^–20^).

Homozygous mutant (var/var) animals showed a slightly higher mean genomic inbreeding coefficient (∼9%) compared with homozygous wildtype and heterozygous animals (∼8.6%), indicating a modest increase in inbreeding in affected individuals (Supplementary Fig. S1).

### 3.5 Clinical findings suggest a novel form of LIPC-associated postural proprioceptive deficits

The clinical presentation of case 1 closely resembled that of the 11 additional cases identified based on their *LIPC* homozygous genotype. Therefore, clinical findings are presented collectively for all 12 cases, with case-specific details highlighted where relevant. All examined animals were female BS cattle, with a mean age of 2.4 ± 0.2 years; four were heifers and eight were first-lactation cows.

**Fig. 2:**
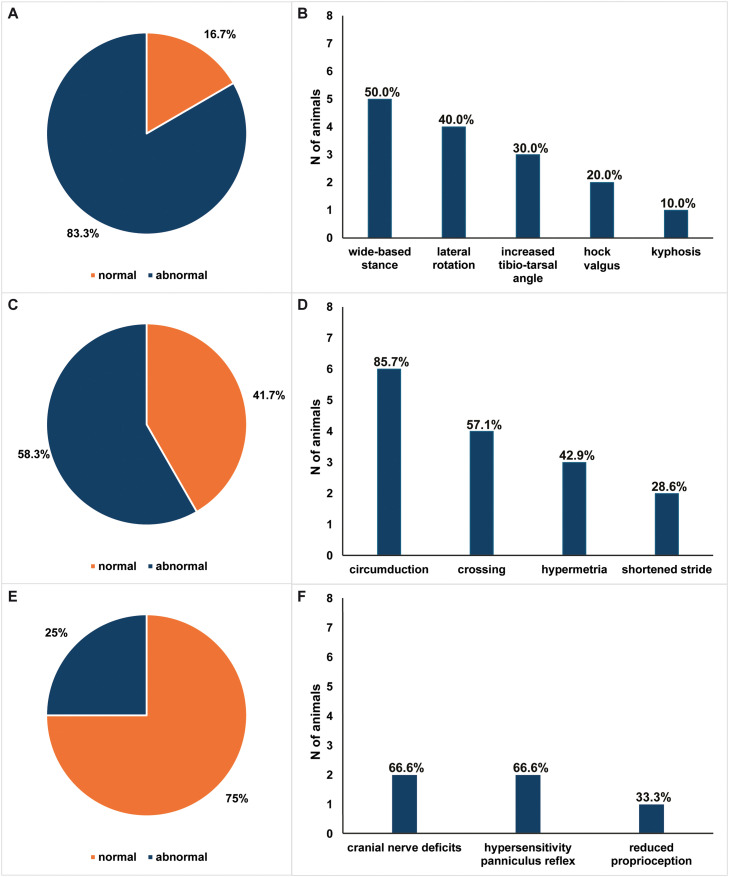
Distribution of neurological clinical signs observed in *LIPC*-homozygous Brown Swiss cattle.

**Fig. 3:**
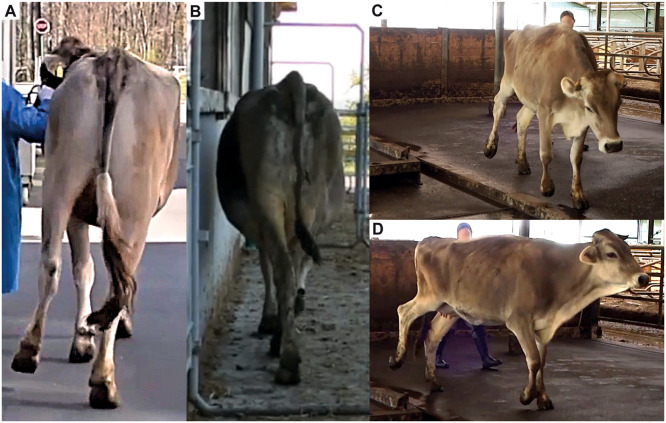
Clinical presentation of ataxic gait in *LIPC*-homozygous Brown Swiss cattle.

The main clinical findings are summarized in Table 2 (see Supplementary Table S2 for more details). Of the 12 animals examined, 83.3% showed postural abnormalities and 58.3% exhibited gait abnormalities consistent with ataxia (Fig. 2. A). The postural abnormalities included a wide-based stance of the pelvic limbs (50.0%), lateral rotation of the distal thoracic limbs (40.0%), an increased tibio-tarsal angle (30.0%), hock valgus of the pelvic limbs (20.0%) and kyphosis (10.0%) (Fig. 2. B). The gait abnormalities presented as circumduction of the pelvic limbs (85.7%) (Fig. 3. A), crossing of the pelvic or all four limbs (57.1%) (Fig. 3. B), hypermetria of the pelvic limbs (42.9%) (Fig. 3. C and D) and/or a prolonged flight phase with a shortened stride of the pelvic limbs (28.6%) (Fig. 2. C and D; Supplementary Video S1). Additional findings from the neurological examination included cranial nerve deficits (66.6%), panniculus reflex hypersensitivity (66.6%), and reduced proprioception of the pelvic or all four limbs (33.3%) (Fig. 2. E and F). The cranial nerve deficits presented as intermittent nystagmus and exophthalmos with medioventral strabismus.

**Table 2:** Clinical findings in 12 *LIPC*-homozygous Brown Swiss cattle.

Case	Age at examination (years)	Lactation status	Anamnesis	Posture	Gait description	Other findings of neurological exam
1	2.5	Heifer	Gait impairment observed ∼1 month prior to clinical examination	Wide-based stance	Intermittent circumduction of pelvic limbs (HL > HR), intermittent prolonged flight phase with shortened stride, intermittent crossing of the limbs over the midline (thoracic > pelvic); signs exacerbated when blindfolded	Reduced proprioception, intermittent nystagmus
2	2	Heifer	No abnormalities reported	Normal	Normal	NA
3	2	Heifer	No abnormalities reported	Wide-based stance of pelvic limbs; lateral rotation of distal thoracic limbs	Normal	NA
4	2.5	1st lactation cow	No abnormalities reported	Wide-based stance of pelvic limbs	Bilateral intermittent circumduction of pelvic limbs (HL > HR), sporadic hypermetria after stepping over obstacles	NA
5	2.5	1st lactation cow	Gait impairement observed ∼ 6 months prior clinical examination	Oblique pelvis (right ischial tuberosity higher than left), tail base deviated right, wide-based stance of the pelvic limbs, left pelvic limb placed more laterally	Intermittent crossing of the pelvic limbs over the midline, intermittent circumduction of the outside pelvic limb walking on a circle	NA
6	2.5	1st lactation cow	No abnormalities reported	Lateral rotation of both distal thoracic limbs, bilaterally increased tibio-tarsal angle	Bilateral intermittent circumduction of the pelvic limbs (HR > HL), enhanced when walking on a circle	NA
7	2.5	1st lactation cow	Gait impairement observed ∼ 6 months prior clinical examination	Lateral rotation of both distal thoracic limbs, hock valgus, caudal thoracolumbar kyphosis	Intermittent crossing of the pelvic limbs over the midline, prolonged flight phase with shortened stride, intermittent hypermetria of pelvic limbs after prolonged walking and sharp turns	Reduced proprioception, mild right-sided exophthalmos and medioventral strabismus, hypersensitivity in lumbosacral region
8	2.5	1st lactation cow	No abnormalities reported	Lateral rotation of both distal thoracic limbs, hock valgus	Bilateral circumduction of pelvic limbs (HR > HL, especially after prolonged walking), intermittent crossing of pelvic limbs over the midline	NA
9	2.5	1st lactation cow	No abnormalities reported	Normal	Normal	NA
10	2.5	Heifer	No abnormalities reported	Bilaterally increased tibio-tarsal angle	Normal	NA
11	2.5	1st lactation cow	No abnormalities reported	Bilaterally increased tibio-tarsal angle	Normal	NA
12	2.5	1st lactation cow	No abnormalities reported	Wide-based stance of pelvic limbs	Bilateral intermittent circumduction of pelvic limbs, intermediate hypermetria	NA

HL = left hind limb left, HR = right hind limb, NA = not applicable.

A) Distribution of animals affected (abnormal) and unaffected (normal) by postural abnormalities. B) Frequency of animals affected (N = 10) by each type of observed postural abnormality. C) Distribution of animals affected (abnormal) and unaffected (normal) by gait abnormalities. D) Frequency of animals affected (N = 7) by each type of observed gait abnormality. E) Distribution of animals affected (abnormal) and unaffected (normal) by additional neurological deficits. F) Frequency of animals affected (N = 3) by each type of additional neurological deficit. Abbreviations: N = number

A) Case 1 showing circumduction of the left pelvic limb. B) Case 8 exhibiting crossing of the right pelvic limb over the body midline. C) Case 7 showing slight hypermetria of the right pelvic limb. D) Case 7 displaying severe hypermetria of the right pelvic limb.

The results of blood analyses are given in Supplementary Table S3. All parameters of the CBC performed for case 1 were within normal limits.

Plasma biochemistry (performed in 6 animals) was suggestive of dyslipidemia. The mean values revealed hypercholesterolemia (total cholesterol 154 mg/dL ± 81.1 mg/dL; reference values: 80–120 mg/dL), reduced HDL cholesterol (47.8 mg/dL ± 8.7 mg/dL; reference values: ∼66–72 mg/dL) and hypertriglyceridemia (18 mg/dL ± 12.7 mg/dL; reference values: 0–14 mg/dL).

Follow-up interviews conducted three years after first presentation with five owners (cases 1, 4, 5, 7, and 11) revealed that all affected animals had been culled (one euthanized, four slaughtered) at a mean age of 4.1 ± 1.1 years. Reasons for premature culling included poor fertility and/or low milk yield. The euthanized animal had been unable to stand due to progressive gait deterioration. Further follow-up details are available in Supplementary Table S2.

Overall, the clinical signs and biochemical findings were compatible with a novel form of postural proprioceptive deficits in *LIPC*-homozygous BS cattle, associated with a tendency toward dyslipidemia.

### 3.6 Pedigree analysis support possible recessive inheritance

**Fig. 4:**
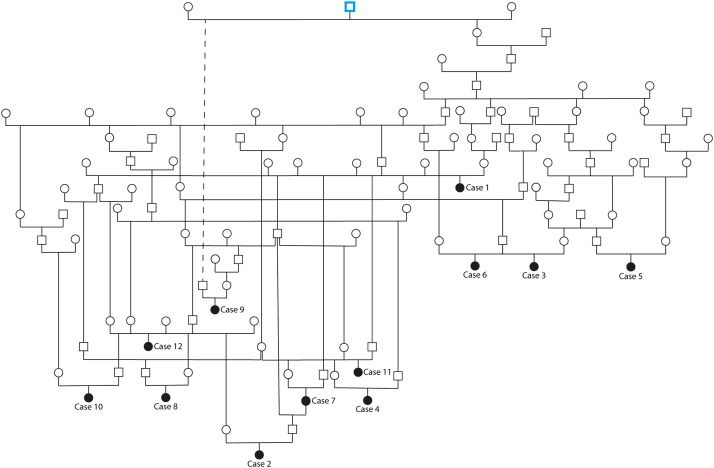
Pedigree of the 12 *LIPC*-homozygous Brown Swiss cases. Affected individuals (case 1 to 12) are homozygous for the *LIPC* variant and are represented by black-filled circles, demonstrating familial clustering consistent with a monogenic autosomal recessive mode of inheritance. The common ancestor of the 12 cases is highlighted with a blue-outlined square*.* Additional pedigree information linking the sire of case 9 to the common ancestor was reduced to a dashed line for clarity and can be found in Supplementary Fig. S2.

Pedigree analysis revealed that the 12 clinically studied *LIPC*-homozygous BS cases shared a common ancestor, an artificial insemination sire born in the year 1966 (Fig. 4, Supplementary Figure S2). The presence of this shared ancestor across all examined affected individuals, combined with multiple loops of inbreeding in the pedigree, further supports the hypothesis of a recessive mode of inheritance.

Some examples regarding the inbreeding, cases 2, 6, 7 and 11 each show one or more inbreeding loops in the 2nd and 3rd or 3rd and 4th generation. Notably, case 2 is a granddaughter of case 7, further strengthening the evidence for inheritance through related carriers.

## 4 Discussion

This study identified a previously unrecognized autosomal recessive form of juvenile-onset postural proprioceptive deficits in BS cattle, which is associated with a pathogenic variant in the *LIPC* gene. A heterozygous spontaneous dominant *de novo* mutation was also considered in the index case but deemed unlikely due to the absence of pathogenic heterozygous variants rare to the affected sequenced animal. Genome-wide filtering under the assumption of a recessive mode of inheritance revealed a single rare protein-changing homozygous variant in the *LIPC* gene, which is predicted to impact the lipase domain. Through population-wide targeted genotyping in the Swiss dairy cattle population, it was possible to validate the variant in a series of clinically examined cases. The variant segregated in the BS population with an allele frequency of 17%, accompanied by a significant deviation from the Hardy-Weinberg equilibrium, suggesting potential selection against homozygosity as reported for other deleterious alleles (Häfliger et al., 2021; Neamatzadeh et al., 2024; VanRaden et al., 2011). It is hypothesized that this variant could also be associated with embryonic, fetal or perinatal lethality in homozygotes and therefore explaining the significant deviation from the Hardy-Weinberg equilibrium (Häfliger et al., 2021; Neamatzadeh et al., 2024; VanRaden et al., 2011). It could be speculated that unknown additional variants in modifying genes might suppress the expression of this condition. Pedigree analysis further supported recessive inheritance, with several cases showing inbreeding loops and all tracing back to a common ancestor. These findings highlight the importance of genomic surveillance and targeted breeding strategies to manage and reduce the prevalence of this deleterious variant in the BS population.

The onset of the disorder was estimated at 2.5 years of age, with most affected animals exhibiting consistent postural proprioceptive deficits and gait abnormalities indicative of ataxia, defined as a proprioceptive dysfunction leading to inconsistent limb movement or placement (Mayhew & Mackay, 2022). Common postural findings consistent with proprioceptive deficits included a wide-based stance, increased tibio-tarsal angle, and kyphosis. Gait abnormalities were characterized by limb crossing over the midline in both thoracic and pelvic limbs, as well as circumduction and hypermetria, primarily affecting the pelvic limbs. Additional neurological signs included nystagmus, strabismus, increased panniculus reflex, and reduced proprioception in a total of three animals. Collectively, these neurological findings are consistent with postural proprioceptive deficits and subconscious proprioceptive ataxia (Mayhew & Mackay, 2022). This form of ataxia results from a disruption of the proprioceptive pathways of the spinal cord and or brainstem, and less commonly of sensory nerves (Mayhew & Mackay, 2022). While hypermetria is typically associated with cerebellar disease, its presence alongside nystagmus, without accompanying intention tremor, suggests a lesion in the spinocerebellar tract rather than primary cerebellar dysfunction (Mayhew & Mackay, 2022). In summary, the neurological clinical presentation suggests a lesion most likely localized to the cervical spinal cord. Considering the cranial nerve deficits seen in two of the cases, the caudal brainstem is also a plausible localization, or those two animals could suffer from an additional, potentially unrelated lesion or disorder. Confirmation of this interpretation would require future histopathological investigations of affected animals.

The *LIPC* gene encodes for the hepatic lipase (HL), an enzyme synthesized and secreted by hepatocytes (Tilly-Kiesi et al., 2004). In humans extensive research has reported, that HL plays a key role in lipid metabolism by hydrolyzing triglycerides and phospholipids and is involved in the conversion of very low-density lipoprotein (VLDL) and intermediate-density lipoprotein (IDL) to low-density lipoprotein (LDL), and the transport of high-density lipoprotein (HDL) to the liver (Connelly, 1999; Tilly-Kiesi et al., 2004). LDL transports cholesterol to extrahepatic tissues, while HDL carries cholesterol in the reverse direction, from extrahepatic tissues to the liver, where it can be excreted in the form of bile acids (Norum et al., 1983). In humans, recessively inherited variants in the *LIPC* gene can impair the secretion of HL into the bloodstream or reduce its enzymatic activity, resulting in hepatic lipase deficiency (OMIM 614025) (Tilly-Kiesi et al., 2004). The deficiency has been described to cause dyslipidemia and changes in the composition of lipoproteins in homozygous individuals (Tani et al., 2016; Tilly-Kiesi et al., 2004). The main biochemical changes observed include hypertriglyceridemia, elevated HDL cholesterol, and either increased or decreased LDL cholesterol levels (Tani et al., 2016; Tilly-Kiesi et al., 2004). In the cattle studied here, we observed hypertriglyceridemia similar to that reported in human HL deficiency but notably accompanied by decreased HDL cholesterol levels.

In humans, dyslipidemia, in particular low HDL cholesterol, has been linked to atherosclerosis which is understood to increase the risk for cardiovascular and neurological diseases in humans (Aarli, 1968; Linton et al., 2015). Two studies linked *LIPC* variants to an increased risk for ischemic stroke (Hindorff et al., 2008; Pan et al., 2023). By analogy, it can be hypothesized that comparable ischemic or vascular mechanisms may occur in *LIPC*-homozygous cattle with dyslipidemia, potentially contributing to the neurological deficits observed in the present study.

Interestingly, *LIPC* is highly expressed in the central nervous system and is predicted to interact with 13 different genes including *APOB, LMF1* and *TMEM59 (**Baldarelli* et al.*, 2021**;* *Oughtred* et al.*, 2021**).* Notably, *APOB* and *LMF1,* similarly to *LIPC,* are involved in cholesterol metabolism (Devaraj et al., 2025; Packard et al., 2000). In Holstein cattle, *APOB* is associated with cholesterol deficiency (OMIA 001965-9913) (Menzi et al., 2016), and in humans with the recessively inherited hypobetalipoproteinemia (OMIM 615558) (Lee & Hegele, 2014). Disorders related to *APOB* in both bovines and humans are characterized by marked hypocholesterolemia, which contrasts with the hypercholesterolemia observed in the postural proprioceptive deficits -affected BS cattle (Lee & Hegele, 2014; Mock et al., 2016). Notably, *LMF1* has been associated with lipase deficiency in humans (OMIM246650), characterized by lipoprotein lipase and hepatic triglyceride lipase deficiencies, leading to severe hypertriglyceridemia (Péterfy et al., 2007). Interestingly, the *LIPC*-homozygous BS cattle also showed hypertriglyceridemia, suggesting a possible functional link between *LIPC* and *LMF1* in the lipid metabolism (Péterfy, 2012). Additionally, homozygous *Lmf1* mutant mice exhibit neurological deficits including decreased locomotor activity (MGI1856820) (Baldarelli et al., 2024). Finally, *Tmem59* homozygous mutant mice show abnormal dendrite morphology, synapse morphology, neuron physiology and excitatory postsynaptic currents (MGI6158675) (Liu et al., 2018). Similarly, the affected *LIPC*-mutant BS cattle exhibit proprioceptive ataxia and other motor coordination deficits, potentially reflecting comparable disruptions in neuronal connectivity or synaptic function.

Taken together, the function, expression profile, molecular interactions, and functional annotations of the *LIPC* gene suggest a broader biological role in pathways relevant to the development of neurological disorders. Although the identified missense variant in *LIPC* has not yet been functionally characterized, it may contribute to interactions involving key genes such as *APOB, LMF1*, or *TMEM59*, potentially participating in the pathogenesis of the observed phenotype in BS cattle.

Given the high variant allele frequency in the BS population, we recommend incorporating the *LIPC* variant into routine genotyping to enable informed selection and therefore avoid risk matings of heterozygous carriers. Additionally, our findings support the inclusion of *LIPC* in the panel of candidate neurogenes implicated in mammalian neurological disorders.

Study limitations included missing blood biochemistry profiles from six animals. For the six available blood biochemistry profiles, it should be noted that HDL cholesterol is a parameter rarely ever investigated in cattle thus there are no official reference values from the laboratory where the analyses were performed. Additionally, case 1 was the only animal assessed under hospital conditions, while the remaining 11 were examined on-farm under less controlled circumstances. For example, blindfolding as part of the neurological examination was not feasible on farm. No histopathological examinations were conducted, leaving the suspected clinical neurolocalization unconfirmed. Follow-up assessments were based solely on owner-reported information, as repeat clinical evaluations were not possible. Several limitations of the genomic methodology should be acknowledged. These include the use of a reference genome with known gaps instead of the newer T2T references, the absence of a WGS trio approach to evaluate the presence of spontaneous dominant *de novo* mutations, and the inherent limitations of short-read WGS, such as reduced sensitivity for structural variants and complex repetitive regions as well as potential errors in read alignment and variant calling. Differences in the frequencies of *LIPC* genotypes were observed between the various genotyping arrays used in this study. This could be due to technical reasons, such as signals from genotyping being lower in intensity for homozygous animals than for heterozygotes, resulting in unclear clustering.

## 5 Conclusion

This study describes a previously unrecognized Mendelian disorder characterized by the juvenile-onset of neurological abnormalities in BS cattle, which is associated with a protein-changing, single-nucleotide variant in the *LIPC* gene. Most affected homozygous animals exhibited postural proprioceptive deficits and ataxia accompanied by dyslipidemia. Genetic analyses support a recessive mode of inheritance, with a missense variant in the *LIPC* gene identified as a candidate causal allele.

Given the relatively high frequency of the variant allele in the BS population and its apparent negative impact on animal health and performance, consideration of this variant in routine genotyping and breeding programs could help to reduce the prevalence of the disorder.

Further functional and histopathological studies are necessary to clarify the molecular and pathological mechanisms underlying the observed phenotype, thereby proving the potential causality of the proposed variant. The condition has been registered in the OMIA database (OMIA003023–9913; omia.variant:1842), with ATX proposed as the abbreviation for this disorder.

## Data and model availability statement

The WGS data are available under the study accession no PRJEB18113at the European Nucleotide Archive (www.ebi.ac.uk/ena; SAMEA5714976 is case 1 and SAMEA8565028 is case's 1 sire).

## Funding/ financial sources

This study was financially supported by the Arbeitsgemeinschaft Schweizerischer Rinderzüchter (ASR), Zollikofen, Switzerland, the Federal Office for Agriculture (FOAG), Bern, Switzerland. Joana Jacinto is supported in part by the Faculty Clinical Research Platform (FCRP) of the Vetsuisse Faculty of the University of Bern.

## Ethical statement

All animals in this study were examined with the consent of their owners and handled in accordance with the Swiss Animal Welfare Act and institutional ethical guidelines (Swiss Federal Food Safety & Veterinary Office, 2024). The SNP array genotypes were generated during population-wide genotyping of Swiss dairy populations for the purpose of genomic selection. Collection of blood samples was approved by the Cantonal Committee for Animal Experiments (Canton of Bern; permit BE94/2022).

## CRediT authorship contribution statement

**Bettina A. Weber:** Writing – review & editing, Writing – original draft, Methodology, Data curation. **Aline Zimmermann:** Writing – review & editing, Methodology, Formal analysis. **Irene M. Häfliger:** Writing – review & editing, Software, Methodology. **Franz R. Seefried:** Writing – review & editing, Software, Methodology. **Mireille Meylan:** Writing – review & editing, Methodology. **Cord Drögemüller:** Writing – review & editing, Funding acquisition, Conceptualization. **Joana Jacinto:** Writing – review & editing, Supervision, Methodology, Investigation, Formal analysis, Data curation, Conceptualization.

## Declaration of competing interest

The authors declare no conflict of interest.

## Appendix Supplementary materials

Supplementary Materials Supplementary Table S1. Detailed description and classification of private protein-changing variants identified in case 1 after the comparison with a global control cohort of 5577 other genomes. Supplementary Table S2. Detailed description of the clinical examination results of 12 *LIPC*-homozygous Brown Swiss cattle. Supplementary Table S3. Results of the blood analysis of 6 LIPC-homozygous Brown Swiss cattle. Supplementary Video S1. The video portrays the three main gait abnormalities: circumduction, crossing and hypermetria. Case 1 shows intermittent circumduction of the left pelvic limb and crossing of the left pelvic limb over the midline. Case 8 shows intermittent circumduction of both pelvic limbs and intermittent crossing of both pelvic limbs over the midline. Case 7 shows intermittent hypermetria of the right pelvic limb. Supplementary Figure S1. Distribution of standardized genomic inbreeding coefficient (GenomicF) values by LIPC genotype (LIPCgt) class. The plots show the density of GenomicF standardized to pedigree-based inbreeding for LIPCgt classes ref/ref (0), ref/var (1), and var/var (2). Black squares indicate the mean and black horizontal lines indicate the median for each class; numeric annotations report the corresponding mean and median values. Supplementary Figure S2. Extended pedigree illustrating the additional ancestral link connecting the sire of case 9 to the common ancestor (blue-outlined square) shared by all 12 homozygous *LIPC* cattle. Image, application 1
