# Supplementary figures and images for: Novel LIPC-related recessive form of postural proprioceptive deficits in Brown Swiss cattle
Source: Vet Anim Sci. 2026 Apr 2;32:100645. doi: 10.1016/j.vas.2026.100645 (PMC13089128; doi:10.1016/j.vas.2026.100645)

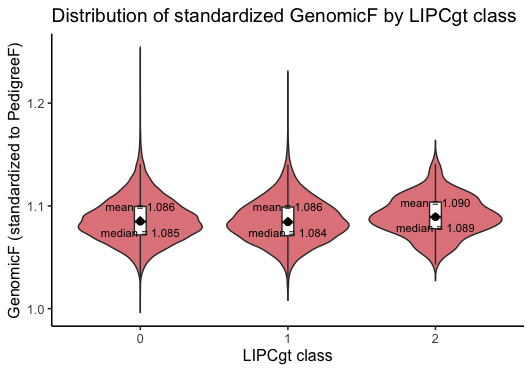

Supplement: Supplementary file 1 — Supplementary Materials Supplementary Table S1. Detailed description and classification of private protein-changing variants identified in case 1 after the comparison with a global control cohort of 5577 other genomes. Supplementary Table S2. Detailed description of the clinical examination results of 12 LIPC-homozygous Brown Swiss cattle. Supplementary Table S3. Results of the blood analysis of 6 LIPC-homozygous Brown Swiss cattle. Supplementary Video S1. The video portrays the three main gait abnormalities: circumduction, crossing and hypermetria. Case 1 shows intermittent circumduction of the left pelvic limb and crossing of the left pelvic limb over the midline. Case 8 shows intermittent circumduction of both pelvic limbs and intermittent crossing of both pelvic limbs over the midline. Case 7 shows intermittent hypermetria of the right pelvic limb. Supplementary Figure S1. Distribution of standardized genomic inbreeding coefficient (GenomicF) values by LIPC genotype (LIPCgt) class. The plots show the density of GenomicF standardized to pedigree-based inbreeding for LIPCgt classes ref/ref (0), ref/var (1), and var/var (2). Black squares indicate the mean and black horizontal lines indicate the median for each class; numeric annotations report the corresponding mean and median values. Supplementary Figure S2. Extended pedigree illustrating the additional ancestral link connecting the sire of case 9 to the common ancestor (blue-outlined square) shared by all 12 homozygous LIPC cattle. [file mmc1.zip › Supplementary_Figure_S1.png]

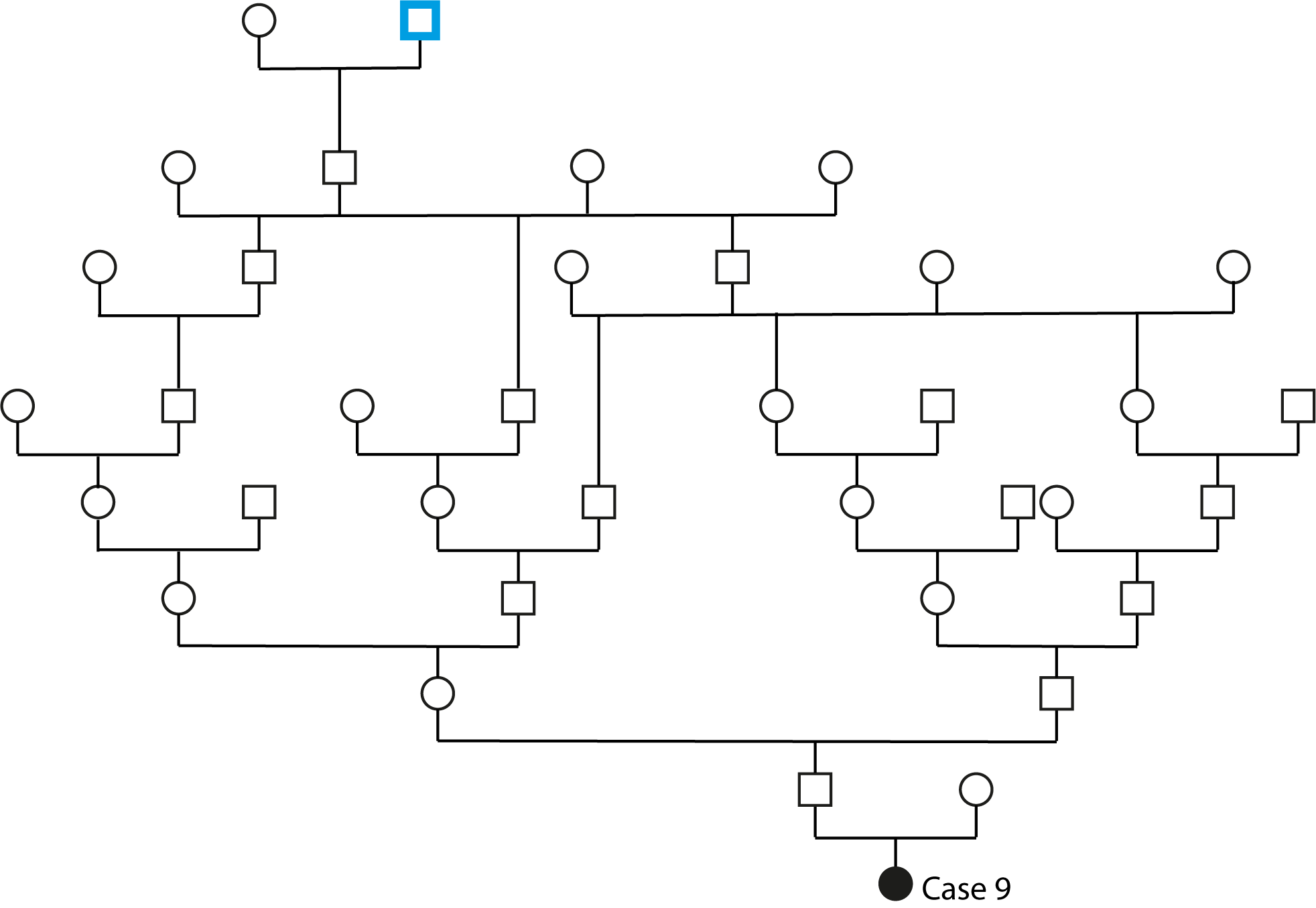

Supplement: Supplementary file 1 — Supplementary Materials Supplementary Table S1. Detailed description and classification of private protein-changing variants identified in case 1 after the comparison with a global control cohort of 5577 other genomes. Supplementary Table S2. Detailed description of the clinical examination results of 12 LIPC-homozygous Brown Swiss cattle. Supplementary Table S3. Results of the blood analysis of 6 LIPC-homozygous Brown Swiss cattle. Supplementary Video S1. The video portrays the three main gait abnormalities: circumduction, crossing and hypermetria. Case 1 shows intermittent circumduction of the left pelvic limb and crossing of the left pelvic limb over the midline. Case 8 shows intermittent circumduction of both pelvic limbs and intermittent crossing of both pelvic limbs over the midline. Case 7 shows intermittent hypermetria of the right pelvic limb. Supplementary Figure S1. Distribution of standardized genomic inbreeding coefficient (GenomicF) values by LIPC genotype (LIPCgt) class. The plots show the density of GenomicF standardized to pedigree-based inbreeding for LIPCgt classes ref/ref (0), ref/var (1), and var/var (2). Black squares indicate the mean and black horizontal lines indicate the median for each class; numeric annotations report the corresponding mean and median values. Supplementary Figure S2. Extended pedigree illustrating the additional ancestral link connecting the sire of case 9 to the common ancestor (blue-outlined square) shared by all 12 homozygous LIPC cattle. [file mmc1.zip › Supplementary_Figure_S2.tif]
